# Composition and Anti-Acetylcholinesterase Properties of the Essential Oil of the Ecuadorian Endemic Species *Eugenia valvata* McVaugh

**DOI:** 10.3390/molecules28248112

**Published:** 2023-12-15

**Authors:** James Calva, Maricarmen Silva, Vladimir Morocho

**Affiliations:** Departamento de Química, Universidad Técnica Particular de Loja (UTPL), Loja 1101608, Ecuador; msilva2@utpl.edu.ec (M.S.); svmorocho@utpl.edu.ec (V.M.)

**Keywords:** Alzheimer, *Eugenia valvata*, essential oil, cholinesterase, enantioselective, gas chromatography

## Abstract

Alzheimer’s disease is a global health problem due to the scarcity of acetylcholinesterase inhibitors, the basis for symptomatic treatment of this disease; this requires new approaches to drug discovery. In this study, we investigated the chemical composition and anticholinesterase activity of *Eugenia valvata* McVaugt (Myrtaceae) collected in southern Ecuador, which was obtained as an essential oil (EO) with a yield of 0.124 ± 0.03% (*w*/*w*); as a result of the chemical composition analysis, a total of 58 organic compounds were identified—representing 95.91% of the total volatile compounds—using a stationary phase based on 5% phenyl-methylpolysiloxane, as analyzed via gas chromatography coupled to mass spectrometry (GC-MS) and flame ionization detection (GC-FID). The main groups were hydrocarbon sesquiterpenes (37.43%), oxygenated sesquiterpenes (31.08%), hydrocarbon monoterpenes (24.14%), oxygenated monoterpenes (0.20%), and other compounds (3.058%). Samples were characterized by the following compounds: α-pinene (22.70%), α-humulene (17.20%), (*E*)-caryophyllene (6.02%), citronellyl pentanoate (5.76%), 7-epi-α-eudesmol (4.34%) and 5-iso-cedranol (3.64%); this research was complemented with an enantioselective analysis carried out using 2,3-diethyl-6-tert-butyldimethylsilyl-β-cyclodextrin as a stationary phase chiral selector. As a result, α-pinene, limonene, and α-cadinene enantiomers were identified; finally, in the search for new active principles, the EO reported strong anticholinesterase activity with an IC_50_ of 53.08 ± 1.13 µg/mL, making it a promising candidate for future studies of Alzheimer’s disease.

## 1. Introduction

Since ancient times, the medicinal use of plants for the treatment or alleviation of various diseases has been replaced by pharmaceuticals, and rural populations also claim that medicinal plant therapy is their preferred method for solving health problems [1,2]. In addition, traditional medicine is currently very important, as it includes herbal medicines, which are used by about 80% of the world’s population for primary health care [3,4]. However, health professionals rarely use herbal remedies; their treatments are based on synthetic pharmaceuticals, even for the treatment of minor illnesses [5].

Ecuador is considered one of the 17 most biodiverse countries in the world, with 18,198 vascular plant species (25% of which are endemic) comprising 91 plant families distributed across three continents and island regions [6]. One of these families is Myrtaceae, belonging to the order Myrtales, represented by about 144 genera and 5500 species in the Neotropics [7].

The *Eugenia* genus is known for its wide range of chemical compounds and comprises around 350 native species [8]; it consists of trees and shrubs predominantly distributed from southern Mexico, Cuba and the Antilles to Uruguay, Argentina, Africa, Southeast Asia and the Pacific [9,10,11]. *Eugenia* offers nutritional and bioactive components; potent antioxidant properties; antimicrobial, anti-inflammatory [12,13,14], analgesic, and antibacterial [15] effects; and cholinesterase activity [16]. On the other hand, various microorganisms and parasites have been studied to determine the effectiveness of *Eugenia* genus; these include pathogenic bacteria as well as viruses such as herpes simplex and hepatitis C. Essential oils (EOs) not only exhibit antimicrobial, antioxidant, antifungal, and antiviral properties but also anti-inflammatory, cytotoxic, insect repellent, and anesthetic properties [17]. Furthermore, many *Eugenia* species contain compounds such as gallic polyphenols, ellagic acyl derivatives [18,19], tannins [20] and flavonol glycosides [15,21]. Primarily distributed in tropical and subtropical regions, they are generally subtropical woody plants with persistent foliage and lysigenous secretory cavities containing essences that produce spices and medicinal substances with biological properties [22]. The species *Eugenia valvata* is a shrub or tree species endemic to Ecuador; it is found in the provinces of Cañar, Carchi, Chimborazo, Imbabura, Loja and Pichincha [23]; in the lowland forests of the Andes; and in the humid vegetation of the inter-Andean region, between 1000 and 3500 m.a.s.l. [7,23].

Preliminary studies have shown that essential oils and their chemical constituents have effects on the central nervous system, including in the treatment of Alzheimer’s disease [24]. This is because the components of EOs are small and lipophilic, which facilitates their movement across the blood–brain barrier [25]. In addition, their characteristic volatility may facilitate their use in inhalation, bypassing the metabolic pathway whose facilitator denatures the active components [26]. Clinical reports also suggest aromatherapy improves memory and alleviates psycho-behavioral symptoms in Alzheimer’s patients [27].

To the best of our knowledge, the present study represents the first report of the chemical composition of *E. valvata* EO and Acetylcholinesterase (AChE) inhibition. Furthermore, it presents this chemical characterization, which may contribute to the correct identification of species from this genus; it also presents the first study of its enantiomeric distribution.

The aim of the present study was to extract and characterize the EO present in the species, and to evaluate the EO from *E. valvata* in terms of its inhibition of the AChE enzyme, in order to identify possible species that could represent new sources of cholinesterase inhibitors for the palliative treatment of Alzheimer’s disease.

## 2. Results

### 2.1. Yield and Chemical Composition

The essential oil obtained from *E. valvata* showed a low yield of 0.124 ± 0.03% (*w*/*w*); a total of 58 constituents were identified, accounting for 95.91% of the total components, which were identified by GC-MS and GC-FID. The compounds’ identification and their abundance, as well as the LRI values in order of their elution, are depicted in Table 1.

**Figure 1:**
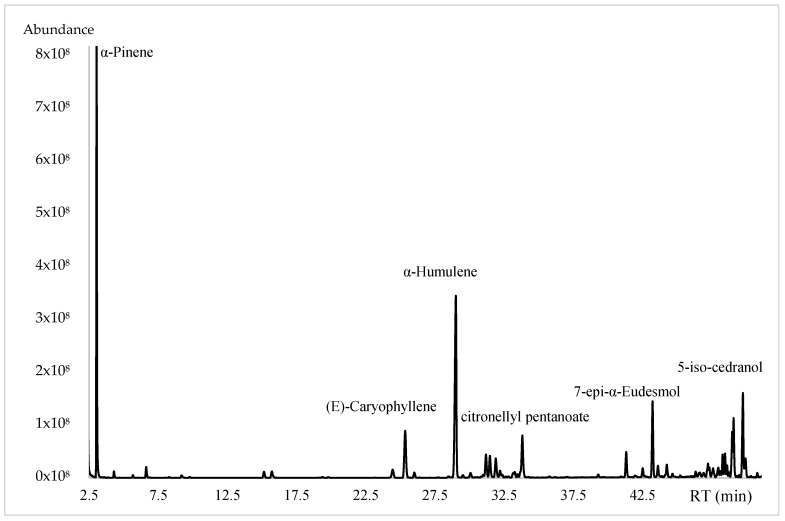
Chromatogram of EO of *Eugenia valvata* on DB5 column.

**Figure 2:**
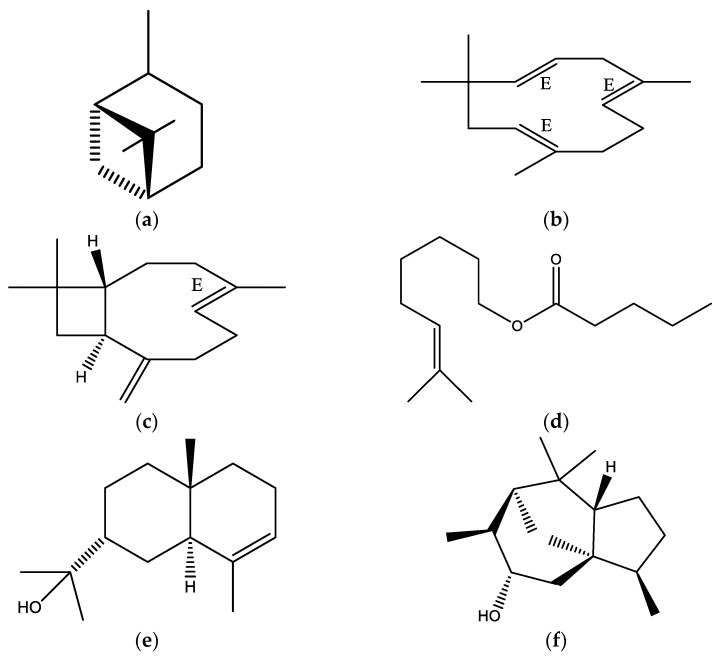
Chemical constituents of *Eugenia valvata* essential oil; (**a**) α-pinene, (**b**) α-humulene (**c**) (E)-caryophyllene, (**d**) citronellyl pentanoate, (**e**) 7-epi-α-eudesmol and (**f**) 5-iso-cedranol.

From the data obtained, the EO showed a complex mixture of several components, predominated by hydrocarbon sesquiterpenes (37.43%) followed by oxygenated sesquiterpenes (31.08%), hydrocarbon monoterpenes (24.14%) and to a lesser extent oxygenated monoterpene (0.20%). Furthermore, the major compounds were (a) α-pinene (22.70%), (b) α-humulene (17.20%), (c) (E)-caryophyllene (6.02%), (d) citronellyl pentanoate (5.76%), (e) 7-epi-α-eudesmol (4.34%) and (f) 5-iso-cedranol (3.64%), as shown in Figure 1 and Figure 2.

### 2.2. Enantiomeric Distribution

The enantiomeric distribution was analyzed for the first time on a chiral stationary phase, considering the retention time of each separated enantiomer using pure standards. Furthermore, hydrocarbon monoterpenes such as α-pinene and limonene, as well as sesquiterpenes such as α-cadinene, were successfully identified. the enantiomeric distribution and enantiomeric excess—which describe how optically pure a mixture is by calculating the purity of the major enantiomer—were assessed, and the results are shown in Table 2.

### 2.3. Cholinesterase Activity

**Figure 3:**
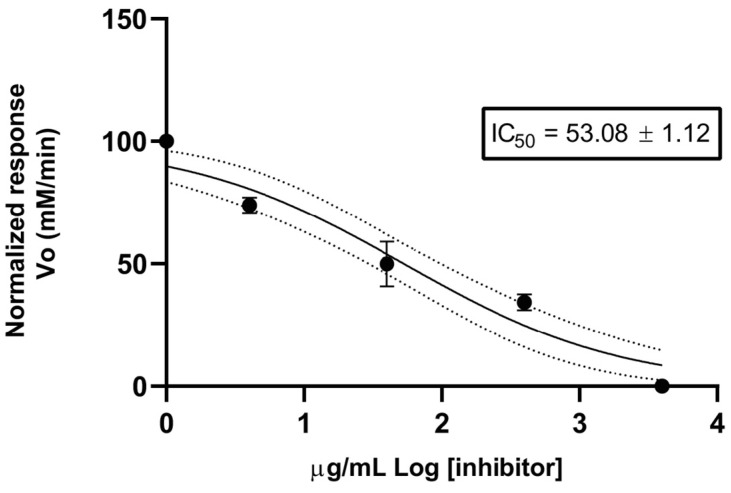
Inhibitory effect plot of *Eugenia valvata* essential oil, against acetylcholinesterase. Data were analyzed_0_, by the non linear regression model, n = 9.

The AChE inhibitory activity of *E. valvata* EO was assessed using a spectrophotometric method. The *E. valvata* EO was assayed for its anticholinesterase potential by measuring the rate against three different concentrations; it showed a moderate inhibitory activity with an IC_50_ value of 53.08 ± 1.13 µg/mL (Figure 3). Furthermore, donepezil hydrochloride was used as a positive control with an IC_50_ value of 12.3 ± 1.35 µg/mL.

## 3. Discussion

Our essential oil yield was significantly lower than that from other species of the same genus. For example, *E. egensis*, *E. flavescens*, *E. polystachya* and *E. patrisii* presented values of 2.5%, 1.0%, 1.0% and 0.7%, respectively [29]; it is important to note that these plants were air-dried, and the yield was similar to that of *E. uniflora* L. (0.13%) [30]. According to the literature, the yield may be related to the influence of variables such as genetic factors, the developmental stage and phenological cycle of seasonal changes, the type of plant material, and other conditions such as geographical distribution [31].

To the best of our knowledge, this is the first report into the essential oil of *E. valvata*, though there are reports on other species of *Eugenia*. This is the case for *E. flavescens*, where oil extraction was performed upon the dried leaves and thin stems, obtaining three isomers of bisabolene as a majority: (E)-γ-bisabolene (35.0%), β-bisabolene (34.7%) and (*E*)-iso-γ-bisabolene (5.1%). In the species *E. egensis*, the main components were 5-hydroxy-cis-calemenene (35.8%), β-caryophyllene (8.9%), trans-cadin-1,4-diene (6.3%), trans-calamenene (6.1%), trans-muurola-3,5-diene (5.9%) and ledol (5.0%) [29]. The EOs of *Eugenia protenta* from Brazil presented three chemical profiles mainly composed of sesquiterpenes such as selin-11-en-4α-ol (14.4–18.3%) and β-elemene (12.3–18.3%) for profile I; germacrene D (15.1–15.6%), bicyclogermacrene (5.8–11.8%), δ-elemene (8.5%) and β-elemene (9.2–12.8%) for profile II; and dimethylxanthoxylin (73.2–83.0%) for profile 3 [32]. Compounds found in *E. umbelliflora* oil—such as viridiflorol (17.7%), β-pinene (13.2%), α-pinene (11.2%), aromadendrene (6.9%) and ledol (4.7%) [33]—and in another EOs from *E. umbelliflora* from southern Brazil—α-pinene and β-pinene—were the main compounds (24.7% and 23.5%, respectively) [34], the only oils similar to our study. Sesquiterpenes predominate in the leaf oil of other *Eugenia* species, and the variability of the compounds is due to the geographical location and genetics of each species, because these factors modify their chemical composition [35].

The main compound in the EO was α-Pinene, a monoterpene of great interest for medical use but also of high industrial and commercial value; it is a bicyclic hydrocarbon consisting of two isoprene units, giving the total formula C_10_H_16_ [36]. α-Pinene is the major secondary metabolite in many coniferous essential oils [37]; it has volatile and hydrophobic properties, a fresh pine scent, and a woody flavor [38].

The biological properties of α -pinene have been extensively studied. However, there is a lack of data regarding the biological effects of its enantiomers. The role of enantioselectivity in determining biological activity is crucial and may explain the conflicting results in the literature [36]. It is generally accepted that the enantiomeric forms have different biological activities. Therefore, careful monitoring of the enantiomeric distribution of (±)-α-pinene in natural products intended for pharmaceutical or other biological purposes is necessary. The biological activities of α-pinene have been extensively studied. However, there is a lack of data regarding the biological effects of the enantiomers. Enantioselectivity plays an important role in the determination of biological activity and may be the reason for the conflicting data in the literature [36]. Enantiomeric forms are known to have different biological activities. Therefore, the enantiomeric distribution of (±)-α-pinene should be monitored in natural products for pharmaceutical or biological use.

The enantiomer (+) α-pinene has been biologically studied and has shown antibacterial [36,39,40], antifungal [36,40,41], antimalarial [42], anti-inflammatory, chondroprotective [43] and neuroprotective [44] activities; on the other hand, (−) α-pinene presents antiviral [42] and neuroprotective [45] activities, which can be contrasted with the cholinesterase effect that the *E. valvata* essential oil possesses due to the presence of these enantiomers. Both enantiomers (with IC_50_ values of 0.40 and 0.44 mM, respectively) have the potential to act as antagonists of acetylcholinesterase and could be of interest in the field of Alzheimer’s disease treatment [44]. Further research is needed to explore the importance of the enantioselectivity of α-pinene, especially in biological activity, as this would help predict its potential therapeutic applications.

The AChE inhibitory activity of *E. valvata* EO has not been reported to date and shows a moderate AChE inhibitory effect with an IC_50_ of 53.08 ± 1.13 μg/mL; this result is better than that of the extract of *Eugenia dysenterica* ex. DC Mart. [16]; the evaluated aqueous extract of *E. dysenterica* showed a moderate inhibitory effect on AChE at a concentration of 100 μg/mL, with an IC_50_ value of 155.20 ± 2.09 μg/mL. In contrast to our study, one study aimed at evaluating the anticholinesterase effects of six *Eugenia* species from Brazil: neither the methanol extracts nor ethyl acetate fractions of *E. handroana*, *E. stigmatosa* and *E. candolleana* were able to significantly inhibit AChE activity; however, in the case of *E. brevistyla*, *E. catharinae* and *E. mattosii*, the methanol extract and ethyl acetate fraction (at 200 μg/mL) were able to significantly inhibit AChE activity (by up to 83%) [46]. It is worth noting that studies have demonstrated the anticholinesterase activity of phenolic compounds, which are abundant in the genus Eugenia [47,48], and to which this activity could be attributed. Researchers have already examined several *Eugenia* species to determine their potential as anti-AChE agents. Other studies on the Eugenia species have shown, for example, that the essential oil of *E. sucata*, which is rich in (E)-caryophyllene (24.6%), shows anti-acetylcholinesterase activity (IC_50_ 4.66 μg/mL) [49]. Furthermore, the oil of *E. verticillata* (syn. *E. riedeliana*), which is rich in valerianol (28.1%), shows an IC_50_ of 67.3 μg/mL [50], and the oil of *E. brasiliensis* with α-pinene (1.77–15.94%), β-pinene (2.98–11.24%), spathulenol (8.10–18.17%), 1-epi-cubenol (4.83–7.46%) and τ-cadinol (10.38–15.30%) shows low antiacetylcholinesterase activity (IC_50_ > 1000 μg/mL) [51]. The sesquiterpene α-humulene, another of the major compounds of *E. valvata* EOs, has been reported to have low AChE inhibition effects (>15 mM) [52].

According to a study by Miyazawa et al. (1997), the terpene hydrocarbon compounds show similar inhibitory activity on AChE to the terpene alcohols [53]. In addition, Aazza et al. (2011) found that the presence of a double bond in the molecular structure of bicyclic monoterpene hydrocarbons resulted in a strong inhibition of AChE activity [54]. On the other hand, Miyazawa et al. (2001) found that the presence of oxygenated groups, especially in ketones, improved the inhibitory effect of sesquiterpenes [55].

Although AChE inhibition may be of great interest in studies of the treatment or slowing of Alzheimer’s disease and other neurodegenerative diseases, and cholinesterase activity could be attributed to the main compounds, it seems more reasonable to attribute the anticholinesterase activity of *E. valvata* EO to an undefined interaction of the enzyme with different components of the oil, and synergistic and antagonistic relationships should be considered.

## 4. Materials and Methods

### 4.1. Plant Material

**Figure 4:**
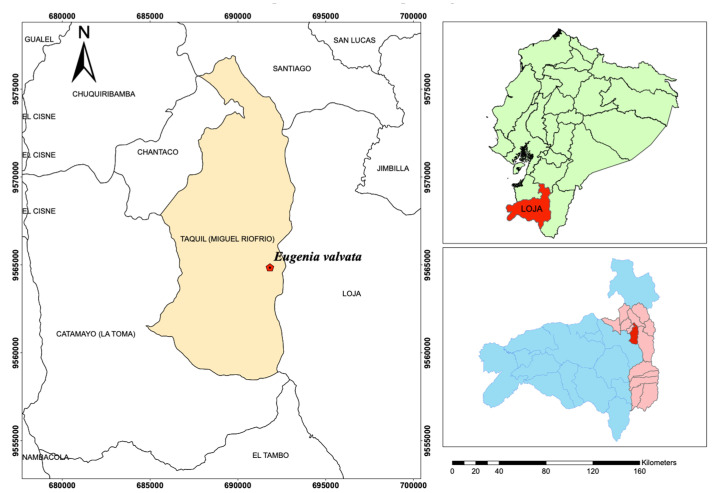
Map of the collection of *Eugenia valvata*; province of Loja, from Ecuador.

The leaves of *Eugenia valvata* in flowering stage were collected in the Chuquiribamba sector of the Loja province (latitude 3°56′06″ S and longitude 79°16′20″ W), on November 2021, as shown in Figure 4. The leaves were cleaned and stored at 0 °C until EO extraction. The identification was conducted by Dr. Nixon Cumbicus, botanist of the Universidad Técnica Particular de Loja (UTPL) and deposited in the herbarium HUTPL with the voucher code 14551; finally, the plant collection was authorized by the government of Ecuador with code MAE-DBN-2016-048.

### 4.2. Essential Oil Isolation

The essential oil was obtained from 2.09 kg of fresh leaves, which were chopped manually and subjected immediately to steam distillation for 3 h at atmospheric pressure, in a Clevenger apparatus. The essential oil obtained was dried over anhydrous sodium sulfate and refrigerated (below −4 °C) until the GC analysis; this process was carried out in triplicate [31].

### 4.3. Identification and Quantification of Essential Oil

Gas chromatography coupled to mass spectrometry (GC-MS) and flame ionization detection (GC-FID) were performed, and the composition of the EO of *Eugenia valvata* and the linear retention indices (LRI) were calculated on the basis of the hydrocarbon standards and via comparison of the mass spectra. Samples were prepared using a ratio of 1:100, with 10 μL of *E. valvata* EO and 990 μL of dichloromethane (HPLC grade, Thermo Fisher Scientific, Waltham, MA, USA).

For the qualitative analysis, a Thermo Scientific gas chromatograph (Trace 1310) coupled to mass spectrometry (ISQ7000) (Thermo Fisher Scientific, Waltham, MA, USA) was performed to determine the chemical composition of the EO. A non-polar DB-5ms based on a 5% phenyl-methylpolysiloxane column (30 m × 0.25 mm, 0.25 μm film thickness) (J & W Scientific, Folsom, CA, USA) was used. Each sample was injected in triplicate in split mode (40:1). The instrument was operated in electronic ionization mode (70 eV), helium gas was used as the carrier gas (1 mL/min) in constant flow, and the furnace operating conditions were as follows: for the first 5 min, the furnace was maintained at an initial temperature of 60 °C; then, an initial temperature ramp of 4 °C/min was applied until 230 °C was reached, and a second temperature ramp of 15 °C/min was applied until a final temperature of 250 °C was reached; this was maintained for 5 min. Both the oil samples and alkanes were injected under the same conditions.

For the quantitative analysis, the same column, DB-5ms (5% phenyl-methylpolysiloxane), was used. The analysis was performed on the same equipment but using a flame ionization detector (FID). Injection conditions were exactly the same as for GC-MS.

For the identification, each EO component was identified by comparing the mass spectrum and the linear retention indices (LRI) against literature results. The LRIs were calculated using a mixture of *n*-alkanes C9–C24 (ChemService, West Chester, PA, USA), and peaks were identified by comparison with mass spectra and retention indices with the NIST 2020 library and ADAMS [28].

### 4.4. Enantiomeric Analysis

Enantiomeric analysis of the essential oil from *Eugenia valvata* was performed for the first time, using GC-MS in a capillary column based on cyclodextrin (2,3-diethyl-6-tert-butyldimethylsilyl-β-cyclodextrin); the injection conditions of both samples and alkanes were the same as for the GC-MS analysis, except for the oven conditions, which were 60 °C for 5 min and a 2 °C/min ramp up to 220 °C; the run time was 90 min.

### 4.5. Cholinesterase Assay

The acetylcholinesterase inhibitory activity for in vitro *E. valvata* EO was evaluated according to Calva et al. [56] using the spectrophotometric method in accordance with the method developed by Ellman et al. [57]. For this, the AChE enzyme from electrophorus electricus (Sigma Aldrich, San Luis, MO, USA) was used in the experiment. In addition, measurements were performed in a microplate spectrophotometer (wavelength: 412 nm) (EPOCH 2, BioTek, Winooski, VT, USA). Anticholinesterase activity was expressed as IC_50_, which represents the concentration of EO required for 50% inhibition. The reference ChE inhibitor donepezil, purchased from Sigma-Aldrich (San Luis, MO, USA), was used as a false positive. IC_50_ values were calculated from the progression curves using Graph Pad Prism software (non-linear regression analysis, PRISM 8.0.1, GraphPad, San Diego, CA, USA). Finally, any increase in absorbance due to spontaneous hydrolysis of ATCh was corrected by subtracting the absorbance at the end of the pre-incubation from the absorbance measured after addition of the enzyme.

## 5. Conclusions

The chemical and enantiomeric composition and AChE inhibitory activity of the essential oil of the endemic Ecuadorian plant *Eugenia valvata* were reported for the first time. The potent ability of *E. valvata* essential oil to inhibit AChE highlights the importance of further investigation into the chemical and biological properties of this plant. The essential oil was characterized by a predominance of sesquiterpenoids; it showed good cholinesterase activity. This activity cannot be specifically attributed to the main constituents, therefore it can be said that our knowledge is insufficient to explain the activity of a specific compound, and further studies on AChE inhibition by monoterpenoids and sesquiterpenoids, as well as their synergistic and antagonistic effects, should be considered.

## Figures and Tables

**Table 1:** Chemical composition of *Eugenia valvata* essential oil from Ecuador.

Compounds	LRI_cal_ ^1^	LRI_lit_ ^2^	% ± SD ^3^	MF
Terpenes				
α-Pinene	932	939	22.70 ± 1.878	C_10_H_16_
β-Pinene	978	974	0.34 ± 0.022	C_10_H_16_
Myrcene	990	988	0.21 ± 0.010	C_10_H_16_
Limonene	1030	1024	0.85 ± 0.054	C_10_H_16_
α-terpineol	1203	1186	0.20 ± 0.003	C_10_H_18_O
Cyclosativene	1365	1371	0.27 ± 0.274	C_15_H_24_
α-cis-Bergamotene	1413	1411	0.25 ± 0.003	C_15_H_24_
(*E*)-Caryophyllene	1419	1417	6.02 ± 0.084	C_15_H_24_
α-trans-Bergamotene	1433	1432	0.44 ± 0.005	C_15_H_24_
α-Humulene	1458	1452	17.20 ± 0.25	C_15_H_24_
β-Santalene	1461	1457	0.20 ± 0.030	C_15_H_24_
β-Chamigrene	1475	1476	0.15 ± 0.006	C_15_H_24_
Widdra-2,4(14)-diene	1478	1481	0.29 ± 0.171	C_15_H_24_
γ-Himachalene	1485	1481	0.20 ± 0.074	C_15_H_24_
β-Selinene	1491	1489	2.20 ± 0.029	C_15_H_24_
δ-Selinene	1498	1492	1.80 ± 0.049	C_15_H_24_
β-Macrocarpene	1503	1499	0.10 ± 0.001	C_15_H_24_
Epizonarene	1505	1501	0.46 ± 0.325	C_15_H_24_
β-Bisabolene	1510	1506	0.58 ± 0.059	C_15_H_24_
α-Alaskene	1512	1512	0.38 ± 0.296	C_15_H_24_
7-epi-α-Selinene	1516	1520	0.37 ± 0.321	C_15_H_24_
δ-Cadinene	1521	1522	1.84 ± 0.057	C_15_H_24_
β-Sesquiphellandene	1527	1521	0.35 ± 0.003	C_15_H_24_
(*E*)-γ-Bisabolene	1529	1529	0.28 ± 0.008	C_15_H_24_
Zonarene	1536	1528	0.40 ± 0.008	C_15_H_24_
α-Cadinene	1541	1537	1.71 ± 0.026	C_15_H_24_
Selina-3,7(11)-diene	1545	1545	1.71 ± 0.027	C_15_H_24_
α-Calacorene	1548	1544	0.08 ± 0.016	C_15_H_20_
Terpenoids and Oxygenated Terpenes				
(*E*)-Nerolidol	1567	1561	0.75 ± 0.020	C_15_H_26_O
Maaliol	1571	1566	0.08 ± 0.004	C_15_H_26_O
Caryophyllene oxide	1589	1582	2.32 ± 0.110	C_15_H_24_O
Gleenol	1594	1589	0.08 ± 0.006	C_15_H_26_O
Guaiol	1602	1600	0.09 ± 0.020	C_15_H_26_O
Geranyl 2-methyl butanoate	1607	1601	0.87 ± 0.029	C_15_H_26_O_2_
trans-β-Elemenone	1614	1602	0.08 ± 0.114	C_15_H_22_O
Citronellyl pentanoate	1620	1625	5.76 ± 0.361	C_15_H_28_O_2_
Eremoligenol	1627	1629	0.59 ± 0.041	C_15_H_26_O
γ-Eudesmol	1635	1630	0.16 ± 0.067	C_15_H_26_O
Cubenol	1637	1645	0.82 ± 0.028	C_15_H_26_O
Agarospirol	1642	1646	1.56 ± 0.091	C_15_H_26_O
Himachalol	1645	1652	2.71 ± 0.154	C_15_H_26_O
Cedr-8(15)-en-10-ol	1649	1650	0.72 ± 0.042	C_15_H_24_O
Selin-11-en-4-α-ol	1650	1658	0.01 ± 0.017	C_15_H_26_O
Valerianol	1654	1656	1.12 ± 0.297	C_15_H_26_O
α-Cadinol	1656	1652	0.08 ± 0.129	C_15_H_26_O
cis-Guaia-3,9-dien-11-ol	1660	1648	1.38 ± 0.072	C_15_H_24_O
7-epi-α-Eudesmol	1667	1662	4.34 ± 0.487	C_15_H_26_O
Intermedeol	1671	1665	2.66 ± 0.152	C_15_H_26_O
5-iso-Cedranol	1684	1672	3.64 ± 0.256	C_15_H_26_O
5-neo-Cedranol	1694	1684	0.45 ± 0.088	C_15_H_26_O
cis-Thujopsenal	1710	1708	0.62 ± 0.079	C_15_H_22_O
(*E*)-Apritone	1716	1708	0.07 ± 0.24	C_15_H_24_O
Other compounds				
2-Heptyl acetate	1041	1038	0.23 ± 0.006	C_9_H_18_O_2_
Methyl octanoate	1129	1123	0.60 ± 0.020	C_9_H_18_O_2_
Heptyl isobutanoate	1216	1246	0.66 ± 0.005	C_11_H_22_O_2_
n-Tetradecane	1403	1400	0.56 ± 0.008	C_14_H_30_
Occidentalol acetate	1678	1681	0.58 ± 0.045	C_17_H_26_O_2_
Longiborneol acetate (=Juniperol acetate)	1680	1684	0.40 ± 0.072	C_17_H_28_O_2_
Hydrocarbon monoterpenes			24.14%	
Oxygenated monoterpene			0.20%	
Hydrocarbon sesquiterpenes			37.43%	
Oxygenated sesquiterpenes			31.08%	
Other			3.06%	
Total identified			95.91%	

^1^ LRI_cal_: Calculated linear retention index; ^2^ LRI_lit_: Linear retention index from Adams [28]; ^3^ SD: mean standard deviation over 3 determinations; %: mean percentage content in the EO over 3 determinations; MF: molecular formula.

**Table 2:** Enantiomeric distribution of *Eugenia valvata* essential oil from Ecuador.

Compound	RT	LRI	ED (%)	e.e (%)
α-(+)-Pinene	4.238	939	58.77	17.53
α-(−)-Pinene	4.282	941	41.23	
(+)-Limonene	8.503	1057	34.23	31.53
(−)-Limonene	8.701	1062	65.77	
α-(+)-Cadinene	37.569	1558	21.63	56.73
α-(−)-Cadinene	37.599	1559	78.37	

RT: retention time; LRI: linear retention index; ED: ennatiomeric distribution; e.e.: enantiomeric excess.

## Data Availability

Raw Data Availability Statements are available from the authors (J.C. and V.M.).

## References

[1] Petrovska B.B. (2012). Historical review of medicinal plants’ usage. Pharmacogn. Rev..

[2] Fokunang C.N., Ndikum V., Tabi O.Y., Jiofack R.B., Ngameni B., Guedje N.M., Tembe-Fokunang E.A., Tomkins P., Barkwan S., Kechia F. (2011). Traditional medicine: Past, present and future research and development prospects and integration in the National Health System of Cameroon. Afr. J. Tradit. Complement. Altern. Med..

[3] Che C.T., George V., Ijinu T.P., Pushpangadan P., Andrae-Marobela K., Badal McCreath S., Delgoda R. (2017). Traditional Medicine. Pharmacognosy, Fundamentals, Applications and Strategies.

[4] Elizagaray B., Castro R. (2013). Cuban scientific production about medicinal plants and natural products from PlantMedCUBA database. Rev. Cub. Plant. Med..

[5] Martínez Y., Gómez L.L. (2013). Social impact of an intervention strategy for the rational prescription of natural medicines implemented in Céspedes during 2011. Rev. Cub. Plant. Med..

[6] Ministerio del Ambiente del Ecuador.2015.Quinto Informe Nacional Para el Convenio Sobre la Diversidad Biológica. Quito. Ecuador..

[7] León S., Valencia R., Pitman N., Endara L., Ulloa C.U., Navarrete H. Libro Rojo de Plantas Endémicas del Ecuador, 2a edición. In Publicaciones del Herbario QCA, Pontificia Universidad Católica del Ecuador 2011. https://ddrn.dk/wp-content/uploads/2018/01/LIBRO_ROJO_de_las_plantas_endemicas_del-1.pdf.

[8] Fischer D.C.H., Limberger R.P., Henriques A.T., Moreno P.R.H. (2005). Essential oils from leaves of two *Eugenia brasiliensis* specimens from southeastern Brazil. J. Essent. Oil Res..

[9] Fernanda Mazine F., Quintino Faria J.E., Giaretta A., Vasconcelos T., Forest F., Lucas E. (2018). Phylogeny and biogeography of the hyper–diverse genus Eugenia (Myrtaceae: Myrteae), with emphasis on E. sect. Umbellatae, the most unmanageable clade. Taxon.

[10] Araujo N.M.P., Arruda H.S., de Paulo Farias D., Molina G., Pereira G.A., Pastore G.M. (2021). Plants from the genus Eugenia as promising therapeutic agents for the management of diabetes mellitus: A review. Food Res. Int..

[11] Wilson P.G., Kubitzki K. (2011). Myrtaceae. The Families and Genera of Vascular Plants.

[12] Da Silva A.P.G., Sganzerla W.G., Jacomino A.P., da Silva E.P., Xiao J., Simal-Gandara J. (2022). Chemical composition, bioactive compounds, and perspectives for the industrial formulation of health products from uvaia (*Eugenia pyriformis* Cambess–Myrtaceae): A comprehensive review. J. Food Compos. Anal..

[13] Öztürk A., Özbek H. (2005). The Anti-Inflammatory Activity of *Eugenia Caryophllata* Essential Oil: An animal model of anti-inflammatory activity. Eur. J. Gen. Med..

[14] Slowing K., Carretero E., Villar A. (1996). Anti-inflammatory compounds of *Eugenia jambos*. Phytother. Res..

[15] Sumono A., Wulan A. (2008). The use of bay leaf (*Eugenia polyantha* Wight) in dentistry. Dent. J. (Majalah Kedokteran Gigi)..

[16] Gasca C.A., Castillo W.O., Takahashi C.S., Fagg C.W., Magallanes P.O., Fonseca-Bazzo Y.M., Silveira D. (2017). Assessment of anti-cholinesterase activity and cytotoxicity of cagaita (*Eugenia dysenterica*) leaves. Food Chem. Toxicol..

[17] Chaieb K., Hajlaoui H., Zmantar T., Kahla-Nakbi A.B., Rouabhia M., Mahdouani K., Bakhrouf A. (2007). The chemical composition and biological activity of clove essential oil, *Eugenia caryophyllata* (*Syzigium aromaticum* L. Myrtaceae): A short review. Phytother. Res..

[18] Park M.K., Park J.H., Shin Y.G., Shin U.K., Kim K.H., Yakhak H. (1997). Chemical constituents of *Eugenia caryophyllata*. Pharmaceut. Soc. Korea.

[19] Son K., Kwon S.Y., Kim H.P., Chang H.W., Kang S.S. (1998). Constituents from *Syzygium aromaticum* Merr. et Perry. Nat. Prod. Sci..

[20] Tanaka T., Nonaka G.I., Nishioka I., Kouno I. (1996). Syzyginins A and B, two ellagitannins from *Syzygium aromaticum*. Phytochemistry.

[21] Schmeda-Hirschmann G. (1995). Flavonoids from Calycorectes, Campomanesia, Eugenia and Hexachlamys species. Fitoterapia.

[22] Bravi V.S., Valle E. (2021). Revisión de constituyentes químicos y propiedades biológicas en especies del género Eugenia (Myrtaceae) Review of chemical constituents and biological properties in species of the genus *Eugenia* (Myrtaceae). Dominguezia.

[23] Jorgensen P., Leon-Yanez S. (1999). Catalogue of the Vascular Plants of Ecuador.

[24] Dobetsberger C., Buchbauer G. (2011). Actions of essential oils on the central nervous system: An updated review. Flavour Fragr. J..

[25] Lahlou M. (2004). Essential oils and fragrance compounds: Bioactivity and mechanisms of action. Flavour Fragr. J..

[26] Ayaz M., Junaid M., Ullah F., Sadiq A., Khan M.A., Ahmad W., Shah M.R., Imran M., Ahmad S. (2015). Comparative chemical profiling, cholinesterase inhibitions and anti-radicals properties of essential oils from *Polygonum hydropiper* L.: A preliminary anti-alzheimer’s study. Lipids Health Dis..

[27] Fung J.K.K., Tsang H.W., Chung R.C. (2012). A Systematic review of the use of aromatherapy in treatment of behavioral problems in dementia. Geriatr. Gerontol. Int..

[28] Adams R.P. (2007). Identification of Essential Oil Components by Gas Chromatography/Mass Spectrometry.

[29] Silva J., Andrade E., Barreto L., Silva N., Ribeiro A., Montenegro R., Maia J. (2017). Chemical Composition of Four Essential Oils of Eugenia from the Brazilian Amazon and Their Cytotoxic and Antioxidant Activity. Medicines.

[30] De Rojas Y.E.C., Lucena M.E., Bustamante M.Y.G., Guerrero K.Y.R., Zambrano R.L.A., De González C.D., Ustaríz F.J., Araujo R.M., Chacón M.R.I. (2022). Composición química y actividad antifúngica del aceite esencial de hojas de *Eugenia uniflora* L. (Myrtaceae). Rev. Cub. Farm..

[31] Calva J., Ludeña C., Bec N., Larroque C., Salinas M., Vidari G., Armijos C. (2023). Constituents and Selective BuChE Inhibitory Activity of the Essential Oil from *Hypericum aciculare* Kunth. Plants.

[32] Zoghbi M., Guilhon G., Sarges F., Pereira R., Oliveira J. (2011). Variabilidad química de los volátiles de las hojas de *Eugenia protenta* McVaugh (Myrtaceae) que crecen de forma silvestre en el norte de Brasil. Bioquím. Sist. Ecol..

[33] Magina M.D.A., Dalmarco E.M., Dalmarco J.B., Colla G., Pizzolatti M.G., Brighente I. (2012). Bioactive triterpenes and phenolics of leaves of *Eugenia brasiliensis*. Quım. Nova.

[34] Apel M.A., Limberger R.P., Sobral M., Henriques A.T., Ntalani H., Verin P., Menut C., Bessiere J.M. (2002). Chemical composition of the essential oils from southern Brazilian Eugenia species. Part III. J. Essent. Oil Res..

[35] Stefanello M.É.A., Cervi A.C., Ito I.Y., Salvador M.J., Wisniewski A., Simionatto E.L. (2008). Chemical composition and antimicrobial activity of essential oils of *Eugenia chlorophylla* (myrtaceae). J. Essent. Oil Res..

[36] Da Silva Rivas A.C., Lopes P.M., De Azevedo Barros M.M., Costa Machado D.C., Alviano C.S., Alviano D.S. (2012). Biological activities of α-pinene and β-pinene enantiomers. Molecules.

[37] Allenspach M., Valder C., Flamm D., Grisoni F., Steuer C. (2020). Verification of chromatographic profile of primary essential oil of *Pinus sylvestris* L. combined with chemometric analysis. Molecules.

[38] Vespermann K.A., Paulino B.N., Barcelos M.C., Pessôa M.G., Pastore G.M., Molina G. (2017). Biotransformation of α-and β-pinene into flavor compounds. Appl. Microbiol. Biotechnol..

[39] De Sousa Eduardo L., Farias T.C., Ferreira S.B., Ferreira P.B., Lima Z.N., Ferreira S.B. (2018). Antibacterial activity and time-kill kinetics of positive enantiomer of α-pinene against strains of *Staphylococcus aureus* and *Escherichia coli*. Curr. Top. Med. Chem..

[40] Ložienė K., Švedienė J., Paškevičius A., Raudonienė V., Sytar O., Kosyan A. (2018). Influence of plant origin natural α-pinene with different enantiomeric composition on bacteria, yeasts and fungi. Fitoterapia.

[41] Nikitina L.E., Startseva V.A., Vakulenko I.A., Khismatulina I.M., Lisovskaya S.A., Glushko N.P., Fassakhov R.S. (2009). Synthesis and antifungal activity of compounds of the pinane series. Pharm. Chem. J..

[42] Van Zyl R.L., Seatlholo S.T., Van Vuuren S.F., Viljoen A.M. (2006). The biological activities of 20 nature identical essential oil constituents. J. Essent. Oil Res..

[43] Rufino A.T., Ribeiro M., Judas F., Salgueiro L., Lopes M.C., Cavaleiro C., Mendes A.F. (2014). Anti-inflammatory and chondroprotective activity of (+)-α-pinene: Structural and enantiomeric selectivity. J. Nat. Prod..

[44] Miyazawa M., Yamafuji C. (2005). Inhibition of acetylcholinesterase activity by bicyclic monoterpenoids. J. Agric. Food Chem..

[45] Yang Z., Wu N., Zu Y., Fu Y. (2011). Comparative anti-infectious bronchitis virus (IBV) activity of (-)-pinene: Effect on nucleocapsid (N) protein. Molecules.

[46] Tenfen A., Vechi G., Cechinel-Zanchett C.C., Lorenzett T.S., Reginato-Couto C.E., Siebert D.A., Vitali L., Micke G., Klein-Júnior L.C., Cechinel Filho V. (2021). Phenolic profile by HPLC-ESI-MS/MS of six Brazilian Eugenia species and their potential as cholinesterase inhibitors. Nat. Prod. Res..

[47] Knez D., Coquelle N., Pišlar A., Žakelj S., Jukič M., Sova M., Mravljak J., Nachon F., Brazzolotto X., Kos J. (2018). Multi-target-directed ligands for treating Alzheimer’s disease: Butyrylcholinesterase inhibitors displaying antioxidant and neuroprotective activities. Eur. J. Med. Chem..

[48] Moneim A.E. (2015). Oxidant/Antioxidant imbalance and the risk of Alzheimer’s disease. Curr. Alzheimer Res..

[49] Lima B.G., Tietbohl L.A.C., Fernandes C.P., Cruz R.A.S., Da Botas G.S., Santos M.G., Silva-Filho M.V., Rocha L. (2012). Chemical composition of essential oils and anticholinesterasic activity of *Eugenia sulcata* spring ex mart. Lat. Am. J. Pharm..

[50] Souza A.D., Lopes E.M.C., Silva M.C.D., Cordeiro I., Young M.C.M., Sobral M.E.G., Moreno P.R.H. (2010). Chemical composition and acetylcholinesterase inhibitory activity of essential oils of *Myrceugenia myrcioides* (Cambess.) O. Berg and *Eugenia riedeliana* O. Berg, Myrtaceae. Rev. Bras. Farmacogn..

[51] Siebert D.A., Tenfen A., Yamanaka C.N., De Cordova C.M.M., Scharf D.R., Simionatto E., Alberton M.D. (2014). Evaluation of seasonal chemical composition, antibacterial, antioxidant and anticholinesterase activity of essential oil from *Eugenia brasiliensis* Lam. Nat. Prod. Res..

[52] Lee D.C., Ahn Y.J. (2013). Laboratory and simulated field bioassays to evaluate larvicidal activity of *Pinus densiflora* hydrodistillate, its constituents and structurally related compounds against *Aedes albopictus*, *Aedes aegypti* and *Culex pipiens* pallens in relation to their inhibitory effects on acetylcholinesterase activity. Insects.

[53] Miyazawa M., Watanabe H., Kameoka H. (1997). Inhibition of acetylcholinesterase activity by monoterpenoids with a p-menthane skeleton. J. Agric. Food Chem..

[54] Aazza S., Lyoussi B., Miguel M.G. (2011). Antioxidant and Antiacetylcholinesterase Activities of Some Commercial Essential Oils and Their Major Compounds. Molecules.

[55] Miyazawa M., Tougo H., Ishihara M. (2001). Inhibition of acetylcholinesterase activity by essential oil from Citrus paradisi. Nat. Prod. Lett..

[56] Calva J., Cartuche L., Castillo L.N., Morocho V. (2023). Biological Activities and Chemical Composition of Essential Oil from *Hedyosmum purpurascens* (Todzia)—An Endemic Plant in Ecuador. Molecules.

[57] Ellman G.L., Courtney K.D., Andres V., Featherstone R.M. (1961). A new and rapid colorimetric determination of acetylcholinesterase activity. Biochem. Pharmacol..

